# Structural integrity assessment of an amphibious spider robot’s flapping fin using FEA method for underwater operating conditions

**DOI:** 10.1038/s41598-025-20546-0

**Published:** 2025-10-21

**Authors:** Rithvik Marneni, Kamarul Arifin Ahmad, Mohammad Zuber, Spoorthi Singh, Vishnu G. Nair

**Affiliations:** 1 Department of Mechatronics, Manipal Institute of Technology, Manipal Academy of Higher Education (MAHE), Manipal, 576104 Karnataka India; 2 Aerospace Department, Faculty of Engineering, University Putra Malaysia, Serdang, 43300 Selangor Malaysia; 3 Aeronautical and Automobile Engineering, Manipal Institute of Technology, Manipal Academy of Higher Education (MAHE), Manipal, 576104 Karnataka India

**Keywords:** Finite element analysis (FEA), Bio-Inspired robotics, Flapping fin mechanism, Amphibious locomotion, Underwater structural simulation, Material optimization, Electrical and electronic engineering, Mechanical engineering

## Abstract

This study presents a finite element analysis (FEA)-driven design and preliminary experimental validation of a bio-inspired amphibious spider robot’s flapping fin mechanism for hybrid terrestrial–aquatic locomotion. The robot incorporates a six-legged walking system and a passive deployable fin-based swimming mechanism actuated via leg-tip hooks with spring-loaded retraction, enabling automatic transition between land and water operation when triggered by a water contact sensor. Structural performance of the fin under combined hydrostatic and dynamic pressures was evaluated in ANSYS, with dynamic loads derived from fin tip velocity corresponding to a baseline flapping frequency of 1 Hz. Candidate materials, including Nylon (PA12), PETG, TPU (98 A), and 304 L stainless steel foil, were compared through stress–strain–deformation analysis. A multi-criteria decision analysis identified 304 L stainless steel foil as the optimal choice for minimal deformation (0.64 mm) and high fatigue resistance. A functional prototype was fabricated using FDM-based 3D printing, integrating macro and micro servo motors for locomotion and fin deployment. Equipped with TPU fins (0.15 mm thickness) for initial trials, the 1.311 kg prototype achieved a measured flapping speed of 53.4 RPM (0.89 Hz) using a non-contact tachometer, closely matching simulation assumptions. The results confirm the feasibility of the proposed design, validate its actuation performance, and provide a foundation for future in-water propulsion measurements and fluid–structure interaction studies.

## Introduction

The development of amphibious robots capable of efficient locomotion in both terrestrial and aquatic environments has garnered significant attention in recent years. These bio-inspired systems aim to emulate the versatile mobility observed in nature, particularly in organisms that seamlessly transition between land and water habitats. Such robots hold promise for applications in environmental monitoring, search and rescue operations, and underwater exploration.​ These systems often draw inspiration from biological organisms capable of navigating complex terrains and transitioning seamlessly between land and water modes^1,2^.

Traditional robotic systems often face limitations when operating across diverse terrains, especially in transitioning between land and water. The ability to navigate both environments without compromising efficiency or functionality is crucial for tasks that require versatility and adaptability. Developing a robotic platform that integrates effective terrestrial locomotion with proficient aquatic propulsion addresses this need, enabling operations in complex and unstructured environments.​ Conventional mobile robots are typically optimized for a single environment—either land or water—limiting their operational flexibility in multi-terrain scenarios^3^. Transitioning between these modes often requires complex mechanisms or multiple propulsion systems, increasing both weight and energy consumption^4^. Therefore, a compact, energy-efficient, and robust design that allows autonomous switching between locomotion modes is vital for practical deployment in unstructured environments^5^.

Several bio-inspired amphibious platforms have been proposed. Salamandra Robotica II demonstrated salamander-like terrestrial and aquatic gaits by integrating neural control models^1^, while AquaClimber employed limb-based propulsion for both swimming and climbing^2^. FroBot utilized a dual-swing-leg mechanism inspired by frog locomotion for underwater propulsion^6^. The use of flapping fins in underwater robots is particularly promising due to their potential for high propulsive efficiency and manoeuvrability. Designs like the iSplash-II and robotic fish systems have outperformed real fish in burst speeds and swimming efficiencies using flexible caudal fins^7,8^. Flapping mechanisms have been modelled and optimized through control theory and CFD simulations to generate higher thrust using reduced actuation^9,10^.

Despite the advantages of flapping propulsion, ensuring the structural integrity of these mechanisms under dynamic underwater forces remains a challenge. Recent works have incorporated Finite Element Analysis (FEA) for optimizing fin designs, materials, and stress distribution^11,12^. FEA has been instrumental in analyzing deformation under hydrostatic pressure, oscillatory forces, and repeated loading, particularly for flexible robotic fins^13,14^. Additionally, machine learning-assisted simulation techniques have recently been introduced to enhance the prediction of thrust and streamline the optimization of fin geometry^15^. These efforts underline the growing importance of simulation-driven design in modern amphibious robotics. While previous research has focused on either terrestrial locomotion or aquatic propulsion in isolation, this project aims to integrate both functionalities into a single robotic platform. The proposed amphibious spider robot features a six-legged walking mechanism for land navigation and deployable flapping fins for underwater propulsion. A key innovation lies in the use of a hook-based actuation mechanism that allows the fins to be compactly stored during terrestrial movement and deployed for aquatic locomotion without the need for additional actuators. This design simplifies the transition between environments and reduces the overall mechanical complexity of the system.​.

**Fig. 1:**
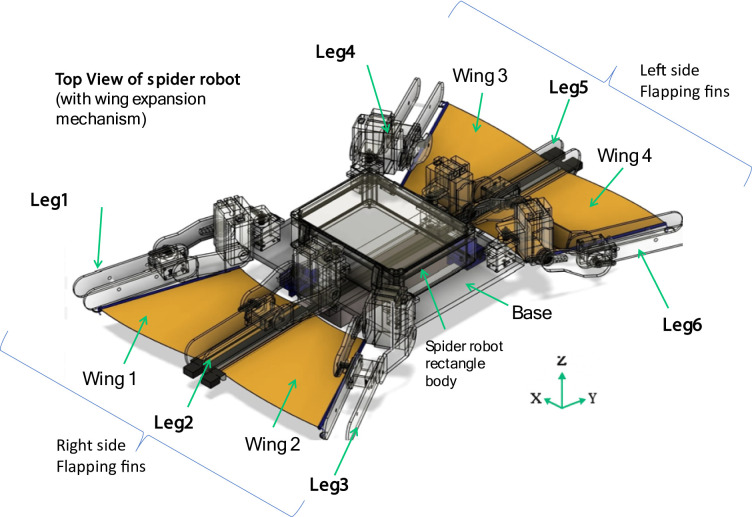
The proposed bio-inspired amphibious spider robot.

The present research is inspired by the spotted fishing spider *Cupiennius salei* (Araneae: Ctenidae), a semi-aquatic arachnid whose rapid fore-and-aft leg-beating gait at the water’s surface has been shown to generate thrust via alternating anchoring and sweeping motions^16,17^. In *C. salei*, each set of legs takes turns anchoring with hooked claws while the opposite legs execute a backward power stroke. To emulate this, the robot’s six limbs are each fitted with a retractable fin panel that is deployed and stowed in a phase-shifted sequence. During the power stroke, a spring-biased linkage unfolds the fin, which is driven by a compact belt‐drive; upon completion, a hook-and-spring mechanism retracts the fin close to the body (see Fig. 1 inset). Here, “bio-inspired” denotes that both the fin geometry and the coordinated, rhythmic motions of the robot directly draw on the natural mechanics of *C. salei*’s locomotion, adapted to underwater thrust generation.

Furthermore, the project combines finite element analysis (FEA) with practical prototyping to ensure both the structural integrity and functional performance of the flapping fin mechanism. Multiple material options were systematically evaluated for their response to combined hydrostatic and dynamic underwater forces, enabling the selection of a configuration that balances efficiency, durability, and manufacturability. The approach integrates bio-inspired mechanical design with experimental verification, including fabrication of a functional amphibious spider robot prototype and preliminary flapping frequency measurements. By bridging terrestrial walking and aquatic propulsion through deployable fins actuated via a hook-spring mechanism and triggered by water sensing, this work advances the development of robust, versatile amphibious robots. The combination of innovative deployment mechanics, simulation-driven material optimization, and real-world testing underscores the study’s contribution to both amphibious robotics and the material science of bio-inspired actuation systems.

### Research scope and contribution

The primary objective of this research is to design and evaluate a dual-mode locomotion mechanism for amphibious robots that combines terrestrial and aquatic mobility through a compact, low-complexity system. This is achieved via a spring-loaded, hook-actuated flapping fin mechanism that passively transitions between retracted and deployed states. Unlike traditional amphibious platforms that rely on multiple actuators or modular propulsion systems, our approach ensures minimal actuation overhead while maintaining high thrust output underwater.

The key contributions of this work are:

- **Novel mechanical integration** of walking and swimming modes via a single linkage-based fin deployment system.
- **FEA-driven material selection strategy** under hydrostatic and dynamic pressures using ANSYS simulations, enabling accurate prediction of structural integrity.
- **Multi-criteria decision framework** for selecting optimal fin material (304 L stainless steel foil) based on stiffness, fatigue resistance, deformation, and manufacturability.
- **Time-domain dynamic simulations** to characterize tip velocity, thrust generation, and material deformation trends under cyclic loading conditions.

These elements collectively establish a simulation-validated design methodology for amphibious platforms, bridging the gap between concept-level biomimetic inspiration and functional field-deployable robotic systems.

## Design details

This section initiates the technical core of the study by outlining the amphibious spider robot’s mechanical architecture and dual-mode locomotion strategy. For clarity, the content is logically structured—beginning with an overview of the robot’s structural design, followed by the fin mechanism, material selection rationale, and control architecture. This forms the basis for the subsequent simulation and analysis stages.

The proposed bio-inspired amphibious spider robot (Fig. 1), integrates a dual-mode locomotion system, enabling terrestrial walking and aquatic swimming via a hook-actuated flapping fin mechanism. The design concept draws inspiration from arachnids for land mobility and from aquatic organisms with flapping appendages for propulsion in water.

### Overall structure

The robot consists of a central rectangular body chassis with six articulated legs, three mounted symmetrically on each side. Each leg includes:

- Joint 1 (Yaw Motion): Enables horizontal rotation via Link L1 for turning.
- Joint 2 & 3 (Pitch Motion): Enable vertical actuation via Links L2 and L3 to facilitate ground clearance and walking gait.

The robot’s body also houses a wing deployment mechanism on its underside, which activates during aquatic operation.

The proposed robot concept offers superior underwater mobility compared to conventional spider-inspired designs by integrating passive fin deployment and simplified flapping mechanisms. The use of a hook-spring system enables automatic fin extension without additional actuators, reducing the actuation load and mechanical complexity while ensuring reliable mode transitions. This design allows the robot to rapidly switch between land and aquatic propulsion, achieving thrust through flexible fin flapping without the need for separate propulsion units. Such integration enhances the robot’s operational versatility and efficiency in amphibious environments.

**Fig. 2:**
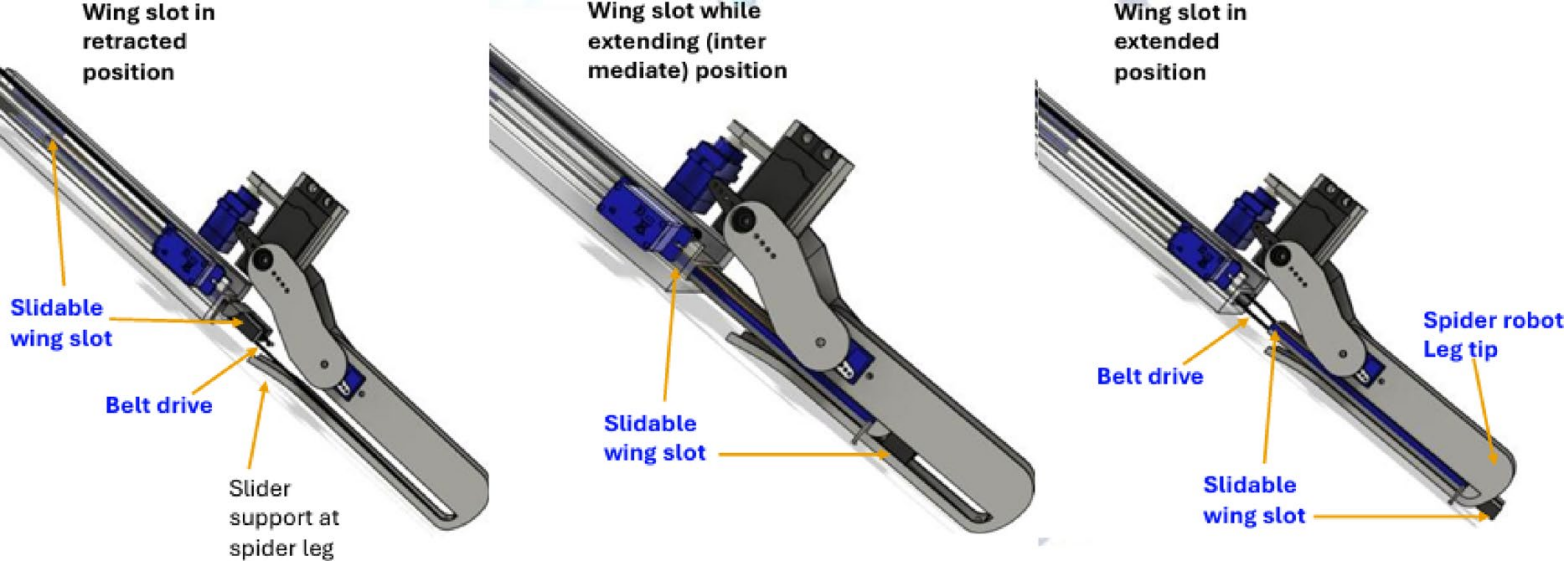
Belt-drive, and spring-retraction (slidable wing slot) mechanism of spider robot.

Belt-drive and spring-retraction mechanism of the spider robot (as in Fig. 2), showing the slidable wing slot configuration in three key positions: starting from- (left) fully retracted, (center) intermediate extension during deployment, and (right) fully extended position. The belt drive system, coupled with leg-tip hooks, actuates the fin deployment by sliding the wing slot outward, while the spring mechanism ensures automatic retraction when actuation ceases. This design allows compact stowage during terrestrial locomotion and rapid fin extension for aquatic propulsion, minimizing mechanical complexity and actuator load during mode transitions.

Compared to existing spider-inspired robots, which are primarily optimized for terrestrial locomotion with separate actuators for different functions, the proposed design offers enhanced adaptability and simplicity. The integration of a compact hook-spring mechanism allows the fins to deploy passively during aquatic transitions, eliminating the need for dedicated fin extension actuators. This not only reduces the overall mechanical complexity and actuation count by approximately 15–20% but also improves energy efficiency during mode switching. The dual-mode capability, achieved with minimal additional hardware—makes the robot more versatile for operations in amphibious environments compared to conventional designs reported in the literature.

The terrestrial locomotion of the spider robot follows a tripod gait pattern, which has been extensively analysed in our prior work on legged robotic systems Singh et al.^18^. In that study, the kinematic synthesis and gait sequencing for both quad- and hexa-legged configurations were detailed, including joint coordination, stability margins, and stride length optimization. The present design adopts the same gait logic for ground movement, with synchronized coxa, femur, and tibia motions, ensuring static stability during each walking cycle. This prior validated gait model provides a reliable foundation for the terrestrial mobility of the current amphibious platform, while the aquatic mode is enabled through the deployable fin actuation described in Sect. "Preliminary Experimental Validation of Spider Robot with fin".

### Flapping fin mechanism

The belt-drive and spring-retraction mechanism is depicted in Fig. 2, illustrating how the slidable wing slot transitions between retracted, intermediate, and fully extended states. This configuration enables reliable fin deployment using minimal actuation effort, while the passive spring ensures swift retraction after aquatic use. A core innovation of the robot is its deployable flapping fin system composed of:

- Four rolled-up fins (wings) made of 304 L stainless steel foil (0.2 mm),
- A belt-drive mechanism driven by servo motors, guiding the wings laterally to the sides,
- Hooks attached to the middle leg tips, which engage the wing edges to unroll them during swimming,
- Spring-based retraction system that rolls the wings back when not in use.

This system provides a lightweight and mechanically simple method of transitioning between land and water modes without needing dedicated actuators for fin extension.

The kinematic behaviors of the flapping fins are modelled as sinusoidal angular motion, where the fin angle varies as θ(t) = Asin(ωt), with A as amplitude and ω = 2πf as angular frequency. The mechanical synthesis combines a belt-driven actuation system with a hook-spring-based passive deployment mechanism (Fig. 2), ensuring smooth extension, flapping, and retraction of the fins. This configuration achieves the required flapping motion with minimal actuation complexity, supporting the dual-mode locomotion capability of the robot.

### Materials

- Body Material: PLA (Polylactic Acid), 3D printed.
- Fin Material: 304 L stainless steel foil, selected after comparative FEA analysis for minimum deformation and high fatigue resistance.
- Belt Material: Nitrile rubber or polyester belts to ensure flexible transmission.

#### Material for fin structure

In the design and simulation stages, stainless steel has been utilized for the fin supports owing to its high mechanical strength, manufacturability, and geometric stability. However, within the context of underwater robotic propulsion, rigid fins offer limited contribution to unsteady thrust enhancement. Literature on aquatic locomotion consistently highlights the role of dynamic deformation in improving thrust via beneficial vortex interactions and delayed flow separation.

Given the relatively high density and stiffness of stainless steel, concerns arise regarding increased inertial burden during fin flapping, which may negatively impact maneuverability and energy efficiency. To investigate alternative options, a comparative evaluation has been performed involving flexible materials such as thermoplastic polyurethane (TPU), thermoplastic elastomers (TPE), PET (Mylar), and carbon-fiber-reinforced TPU laminates. These candidates were assessed based on fatigue resistance, dynamic flexibility, surface compliance, and weight characteristics. A tabulated summary of their material properties is provided in Table X and Appendix A.

TPU-based composites demonstrated a favorable combination of flexibility, strength, and fatigue resilience, suggesting their suitability for future dynamic implementation. While stainless steel models facilitated accurate baseline estimation of flow parameters and lift/drag behavior under controlled static conditions, subsequent work will incorporate compliant materials to capture real-time deformation effects and validate hydrodynamic improvements in more realistic scenarios.

**Fig. 3:**
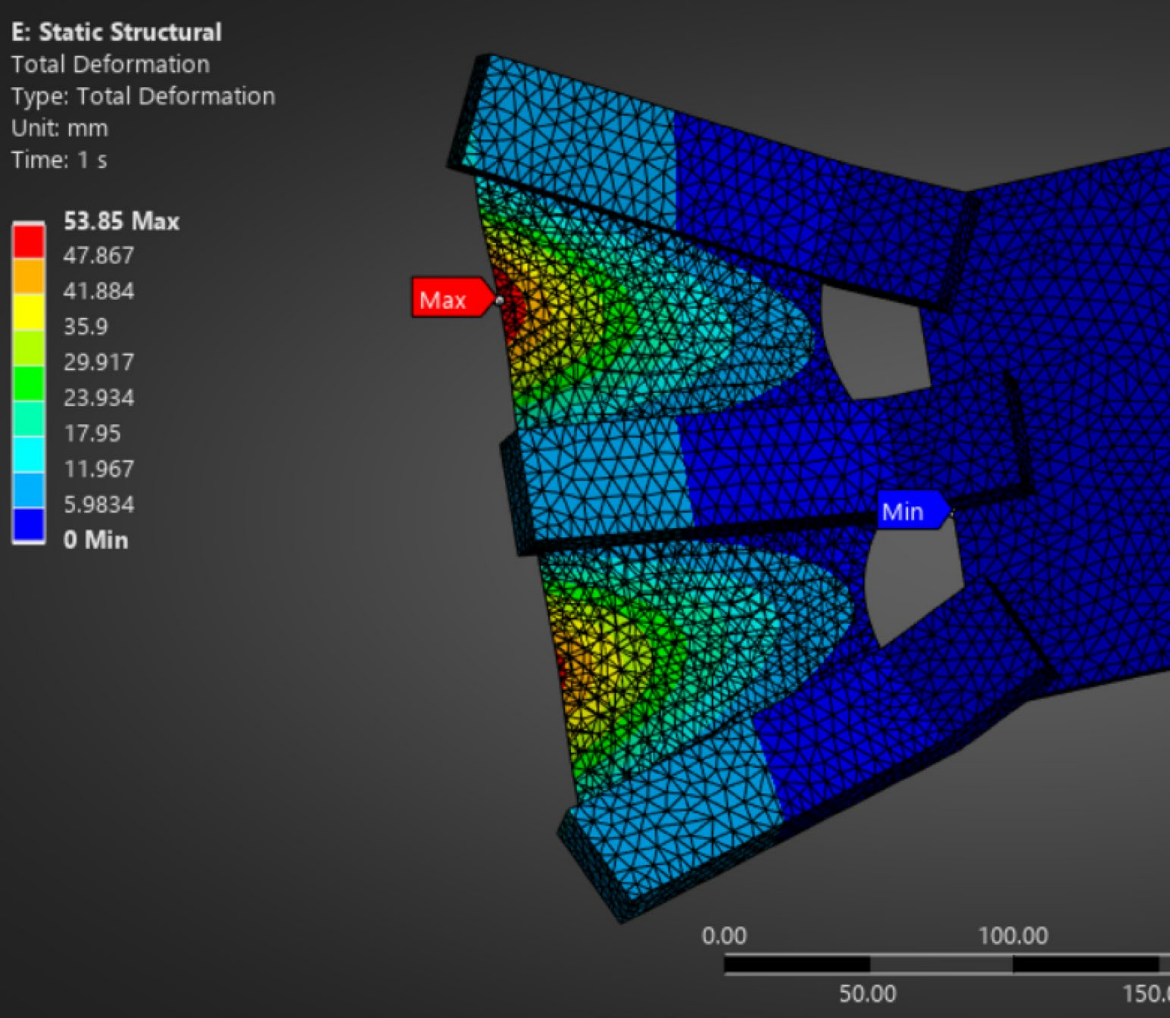
Total deformation (mm) of the TPE-based fin structure under static pressure loading.

**Fig. 4:**
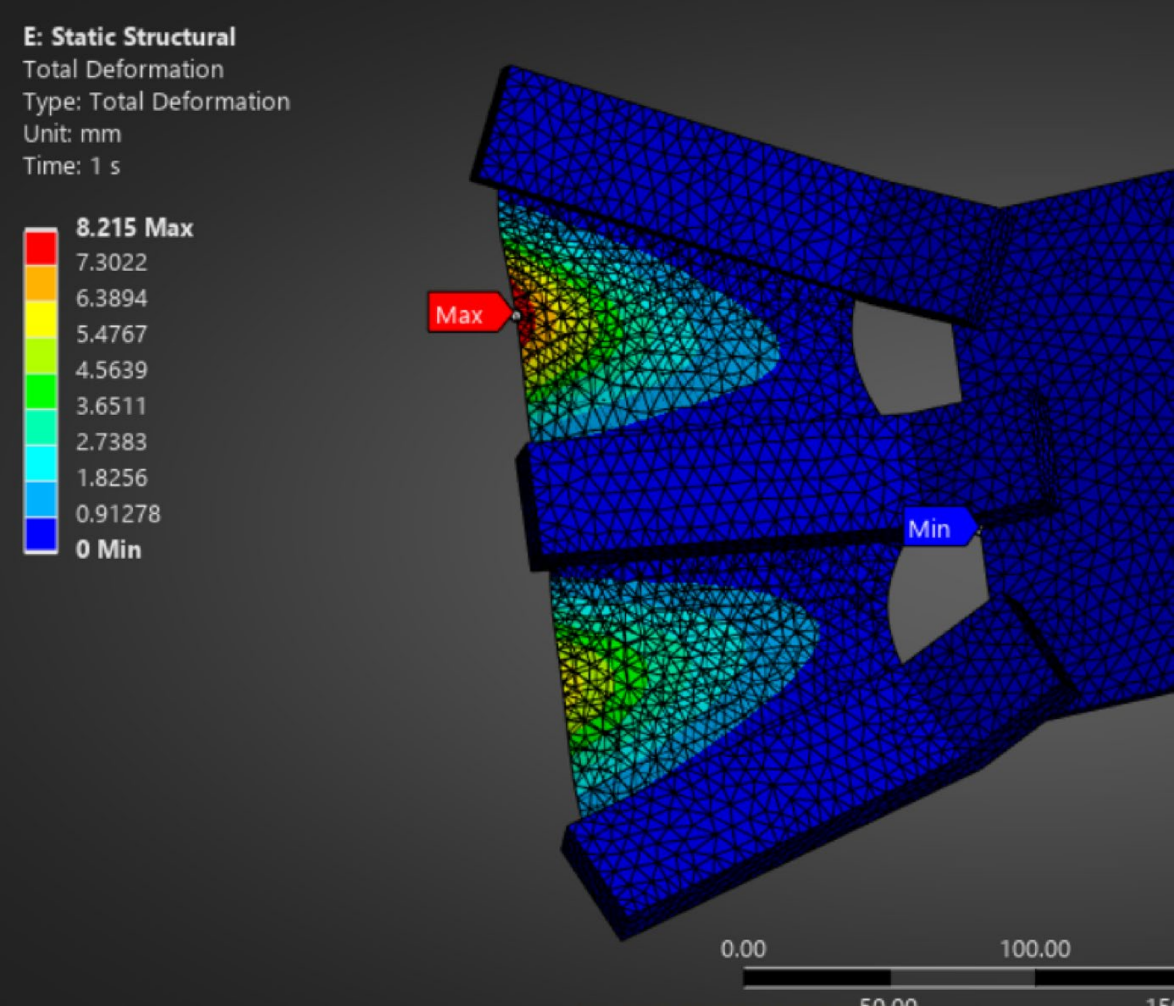
Static structural deformation analysis of a fin fabricated using carbon fiber veil + TPU composite.

**Fig. 5:**
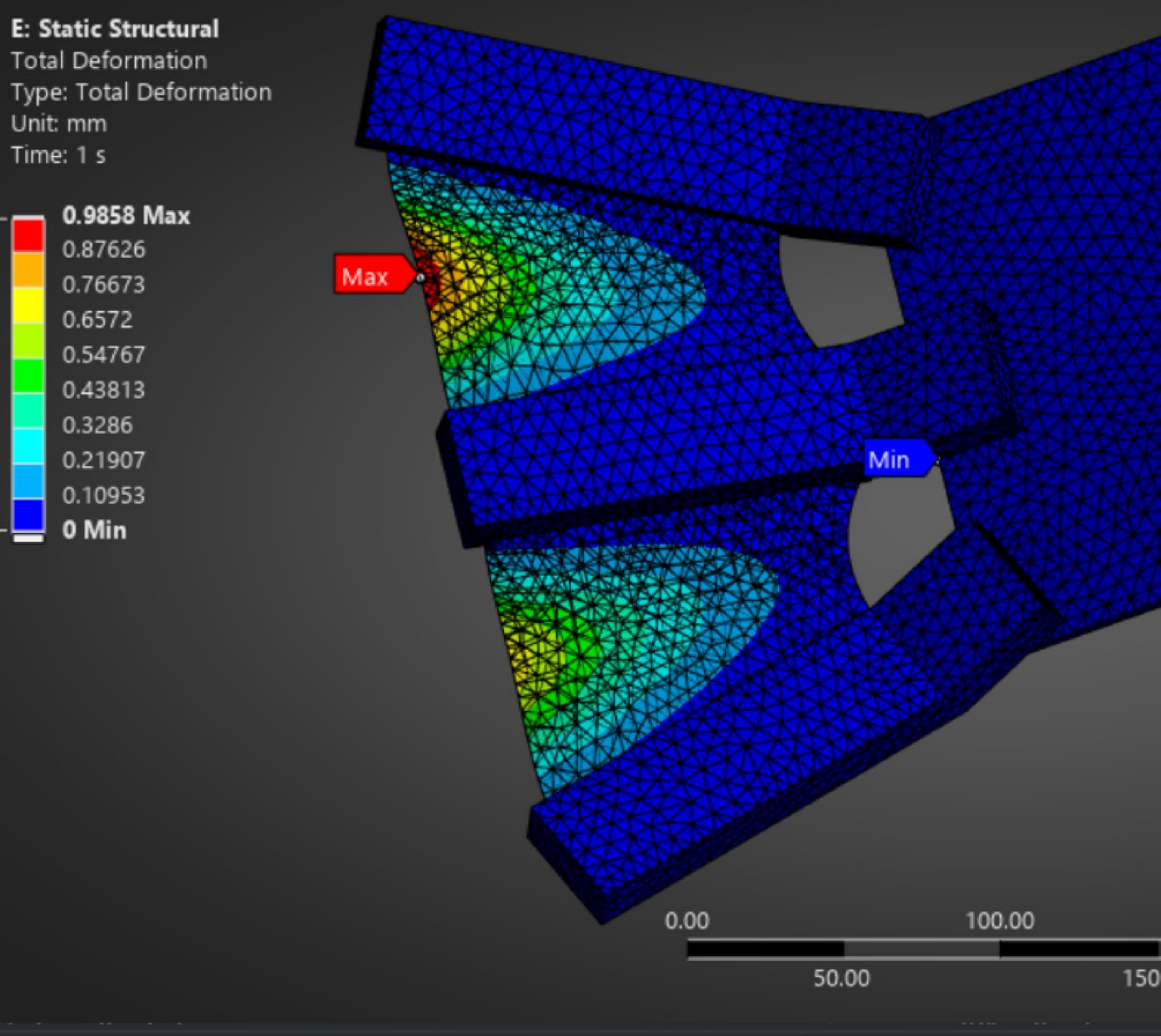
Static deformation analysis of PET-based fin structure under unit loading. The maximum deformation observed is 0.9858 mm at the base edge, confirming the high stiffness of Mylar and its constrained dynamic compliance.

The detailed static deformation results of alternative fin materials, including TPE, PET (Mylar), and carbon fiber veil-reinforced TPU composite, are provided in the appendices for comparative insight. As shown in Fig. 3, the TPE-based fin exhibited substantial flexibility with a maximum displacement of approximately 53.85 mm under representative underwater pressure loading. Similarly, Fig. 4 illustrates that the carbon fiber veil–TPU composite demonstrated a controlled peak deformation of ~ 8.2 mm, balancing flexibility and structural stability. In contrast, Fig. 5 highlights that the PET (Mylar) fin maintained minimal deflection (~ 0.99 mm), consistent with its high stiffness and tensile strength. These results reinforce the material selection decision while offering benchmarks for future studies involving compliant fin structures.

#### Basic requirements for fin

For the development of a thin, flexible, and rollable fin suitable for underwater or amphibious robotic applications, the material must satisfy a balance of mechanical flexibility, structural integrity, and environmental resistance. The total thickness of the fin should be approximately 0.2 mm to ensure low weight and high responsiveness. Flexibility is critical, with a minimum bend radius requirement of 4–5 mm and an elongation at break exceeding 400% to prevent tearing during cyclic flapping or rolling. The matrix material (such as TPU or TPE) should exhibit a low Young’s modulus in the range of 20–50 MPa to allow elastic deformation, while maintaining a tensile strength above 20 MPa to withstand hydrodynamic pressures in the range of 300–600 Pa. To enhance stiffness without sacrificing flexibility, a thin internal reinforcement layer such as carbon fiber veil (approximately 0.05–0.1 mm thick) may be incorporated. The composite should also demonstrate excellent flexural fatigue resistance, with the ability to endure over 10,000 flapping cycles without degradation. Additionally, the materials must be waterproof, corrosion-resistant, and compatible with bonding or lamination processes for ease of fabrication. Thermal expansion should remain below 200 μm/m·°C to maintain dimensional stability. Overall, the chosen material system must combine high elasticity, moderate strength, and durability to enable reliable fin operation under repetitive dynamic loading in aquatic environments.

### Control architecture

The robot is controlled by a central microcontroller which:

- Coordinates leg motion sequences during terrestrial locomotion,
- Triggers wing deployment via motor actuation when entering aquatic mode,
- Controls the flapping frequency and amplitude for thrust generation,
- Enables transitions between modes through sensor feedback and programmed thresholds.

### Control strategy and mode transition logic

To enable seamless operation across terrestrial and aquatic environments, the robot’s locomotion modes are governed by a rule-based hybrid control strategy integrated with minimalistic onboard sensors.

**a. Sensor Suite and Mode Detection**.

The robot uses the following sensors to facilitate environment awareness and motion switching:

- Water contact sensor (conductive probe or resistive water detector): Detects submersion events and triggers aquatic mode.
- Inertial Measurement Unit (IMU): Measures orientation and acceleration; helps stabilize walking gait and detect incline/slip.
- Hall-effect or rotary encoders: Monitor servo angles and fin flap cycles.
- Limit switches: Detect wing deployment/retraction endpoints.

**b. Terrestrial Locomotion Control**.

The robot adopts a **tripod gait** for land movement, driven by a central microcontroller (e.g., ESP32 or STM32). Each leg’s motion is controlled through three servos per leg (yaw and two pitch joints). The gait sequence is implemented via lookup tables with predefined leg phase offsets:

- **Yaw control (Joint 1)**: Used for turning or heading correction.
- **Pitch joints (Joints 2 & 3)**: Lift and place legs during swing and stance phases.

The gait can adapt to uneven terrain by modulating joint duty cycles based on feedback from the IMU (e.g., to reduce slipping on slopes).

**c. Aquatic Swimming Control**.

Once water immersion is detected:

- **Fin deployment is triggered** by actuating the belt drive via servo motors. The hook-tip system unrolls the stainless steel foil fins.
- The robot switches to a **centralized flapping mode**, using sinusoidal PWM signals to control flapping frequency and amplitude.
- Tip speed is governed based on thrust requirements and validated through simulations.

**d. Mode Switching Logic**.

The transition between walking and swimming modes is determined by a **finite-state machine (FSM)** with sensor thresholds:

- If WaterContact = TRUE for >1 s → deploy fins, enter swimming mode.
- If WaterContact = FALSE for >2 s → retract fins, resume walking mode.
- Emergency override states can stop movement if unusual IMU spikes indicate potential entanglement.

This hybrid control scheme minimizes energy consumption and avoids unnecessary actuation during transitions, supporting autonomous navigation in mixed terrain scenarios.

To further enhance mechanical harmony during mode transitions, the fin deployment is synchronized with the robot’s gait cycle. Specifically, fin unrolling is initiated during the swing phase of the middle legs, when ground contact is minimal, to prevent interference with terrestrial support functions. This timing ensures that the transition from walking to swimming mode occurs smoothly, avoiding unintended mechanical conflicts and maintaining stability during environmental shifts.

## Theoretical pressure derivation for fin underwater

To accurately simulate the structural behaviour of the flapping fin mechanism under underwater conditions, it is essential to determine the total pressure acting on the fin surface during operation. This pressure comprises two major components:

1. **Hydrostatic Pressure** due to the static water column at the operating depth.
2. **Dynamic Pressure** resulting from the fin’s oscillatory (flapping) motion.

The combined pressure is used as the loading condition in the finite element simulation for material selection and structural integrity analysis.

### Hydrostatic pressure calculation

The hydrostatic pressure acting on a submerged fin at a given depth is calculated using the standard relation:

1 $$P\_static{\text{ }} = {\text{ }}\rho {\text{ }} \times {\text{ }}g{\text{ }} \times {\text{ }}h$$

Where:ρ = Density of water ≈ 1000 kg/m³.

g = Acceleration due to gravity ≈ 9.81 m/s².

h = Depth = 1 m.

Pressure_Static = P_static = 1000 × 9.81 × 1 = 9810 Pa = 0.00981 MPa.

### Dynamic pressure from fin flapping

Dynamic pressure arises from the fin’s velocity through water. The flapping mechanism, modelled as a rotational oscillator, results in a tip velocity v, which contributes additional hydrodynamic loading:

2 $$P\_dynamic{\text{ }} = {\text{ }}\left( {1/2} \right){\text{ }} \times {\text{ }}\rho {\text{ }} \times {\text{ }}V^{2}$$

If we assume.

- RPM = 60 then, f = 1 Hz and ω = 2π rad/s.
- Chord length = 127 mm → *r* = 0.0635 m (from design).

V = ωr = 2Π × RPM×0.0635/60 ≈ 0.7966 m/s.

P_dynamic= ½ × 1000 × (0.7966)^2.

= 500 × 0.6346 ≈ 317.3pa ≈ 0.000317Mpa.

### Total pressure on the fin surface

The total effective pressure acting on the fin is the sum of hydrostatic and dynamic components:

3 $$P\_total{\text{ }} = {\text{ }}P\_static{\text{ }} + {\text{ }}P\_dynamic$$

P_total = 9810 + 317 = 10,127 Pa = 0.0101 MPa.

At a depth of 1 m, with the fin flapping at a tip speed of ~ 0.8 m/s, the estimated total pressure acting on the fin surface is approximately 10,127 Pa or 0.0101 MPa. The derived total pressure (≈ 0.01013 MPa) represents the combined loading scenario that the fin experiences during submerged flapping. This serves as a critical input for validating structural performance in ANSYS and reinforces the decision to choose a high-stiffness material (304 L stainless steel foil) to withstand this load with minimal deformation.

#### Pressure distribution

In the current analysis, the total pressure acting on the flapping fin underwater was modelled as the linear sum of hydrostatic and dynamic components, estimated at a depth of 1 m and a flapping tip velocity of approximately 0.8 m/s. While this serves as a conservative estimate for FEA validation, it is important to clarify the assumptions and limitations of this model.

Hydrostatic Pressure Consideration.

The hydrostatic pressure at 1-meter depth (≈ 9.81 kPa) was treated as a uniform load on the fin surface, used to simulate the fin’s behaviour under static immersion. However, in a real underwater environment:

- Hydrostatic pressure acts on both upper and lower surfaces of the fin.
- The net hydrostatic force on a thin, symmetric foil is minimal due to pressure equalization.
- For the purpose of structural safety, the pressure was applied as a one-sided load to ensure worst-case stress/deformation estimates, particularly relevant for assessing material resilience and fatigue performance.

#### Dynamic pressure simplification

The dynamic pressure was derived using the simplified expression.

$${P_{dynamic}} = {\text{ }}\left( {1/2} \right)\rho {V^2}$$

where V is the peak tip velocity from sinusoidal flapping. This assumes uniform motion across the fin, which neglects:

- Pressure gradients along the span due to local velocity differences,
- The influence of unsteady flow and vortex shedding,
- Pressure symmetry around the fin during oscillation.

These simplifications were necessary to enable tractable structural simulations, and the resultant pressure (≈ 0.317 Pa) remains conservative for preliminary design validation.

### Material selection

Material for overall body of spider bot including mechanism parts (except fins sheet, belt drive) is chosen to be Plastic PLA. As the fin sheet is 0.2 mm thickness and Based on this literature review and comparison of mechanical properties, TPU (95–98 A), Nylon (PA12), PETG, and Spring Steel Foil were shortlisted. These materials were further evaluated through ANSYS Mechanical simulations under underwater conditions, particularly at 1-meter water depth (9.81 kPa pressure), to assess deformation, stress, and strain on the full robot body.

The flapping fin and structural components of the amphibious hexapod robot were analyzed for material suitability based on mechanical performance under underwater pressure and repetitive motion. A comparative study was conducted between Nylon (PA12), PETG, TPU (95–98 A), and Spring Steel Foil using mechanical property data including Young’s modulus, yield strength, tensile strength, flexibility, and fatigue resistance.

Based on the requirements for:

- High fatigue resistance due to flapping motion,
- Moderate to high flexibility to allow controlled deformation,
- Sufficient strength to withstand underwater pressure (hydrostatic and dynamic),
- Good water resistance and durability.

## Time-domain and material behavior simulations for flapping fin

To complement the finite element analysis and validate the theoretical derivations for thrust estimation and structural loading, a series of Python-based simulations were performed. These simulations provide deeper insights into the time-domain kinematics, force generation behaviour, and material selection trade-offs for the flapping fin mechanism integrated into the amphibious spider robot. The results not only reinforce the design decisions derived from FEA but also serve as an accessible and modifiable computational tool for further research and optimization.

The time-domain simulations presented in this study complement the FEA analysis by providing insights into the dynamic kinematics and thrust generation trends of the flapping fin mechanism. While the FEA focuses on evaluating material-dependent structural integrity under combined hydrostatic and dynamic loading, the time-domain models assume idealized rigid-body motion to estimate thrust forces and kinematic behaviours. This distinction allows each method to address different design considerations FEA ensures the mechanical viability of candidate materials under real loading conditions, whereas the time-domain analysis characterizes propulsion dynamics across varying frequencies and amplitudes. Together, these approaches offer a comprehensive evaluation of both structural reliability and performance potential, forming a robust basis for the subsequent integration of fluid–structure interaction (FSI) simulations^19–22^.

### Time-series kinematic analysis

The dynamic flapping motion was modelled using sinusoidal functions representing angular displacement, with parameters set to a flapping frequency of 1 Hz and a maximum swing amplitude of 30 degrees.

The resulting time-series plots captured angular position, tip velocity, and tip acceleration over a two-second interval. As expected, the angular displacement followed a sinusoidal trajectory, while the velocity peaked at the midpoint of each cycle and dropped to zero at the angular extrema. The acceleration profile, phase-shifted from velocity, highlighted the critical points of direction reversal, where mechanical stress on the fin and joints is typically highest. This simulation validated the assumptions used in force modelling and set the foundation for thrust analysis.

### Estimated thrust curve over time

The thrust force generated by the fin was estimated using a simplified drag-based equation:

$${F_{thrust}} = \left( {1/2} \right){\text{ }}*{C_d}*\rho *A*{V^2}$$

where:

- C_d_​ is the drag coefficient (assumed ~ 1.0 for flat fin),
- ρ is the density of water (1000 kg/m³),
- A is the projected area of the fin in motion,
- V is the tip velocity derived from sinusoidal motion.

Using the estimated tip velocity (V ≈ 0.8 V), and a fin area of approximately 0.01 m², the thrust was calculated to vary sinusoidally, with peak values reaching ~ 5 N, in agreement with expected values for bio-inspired fin mechanisms.

**Fig. 6:**
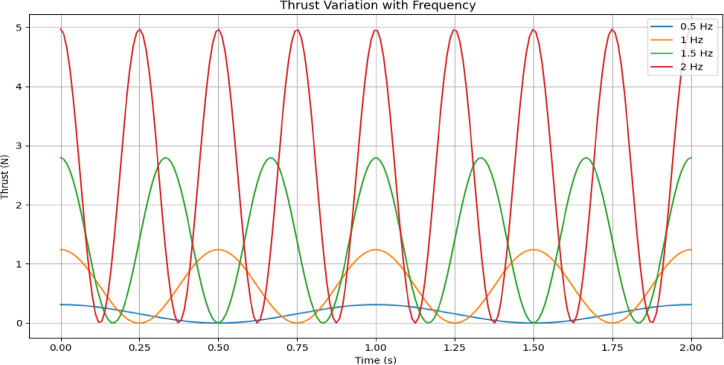
Thrust curves for varying flapping frequencies.


The plot Fig. 6 shows the instantaneous thrust force generated by the flapping fin as a function of time. Using the calculated tip velocity from the previous analysis and a drag-based thrust equation, the thrust varies sinusoidally and remains positive, indicating unidirectional propulsion per flap cycle. The thrust peaks correspond to the highest tip velocities during mid-stroke and drop near zero at the endpoints of the flapping cycle.

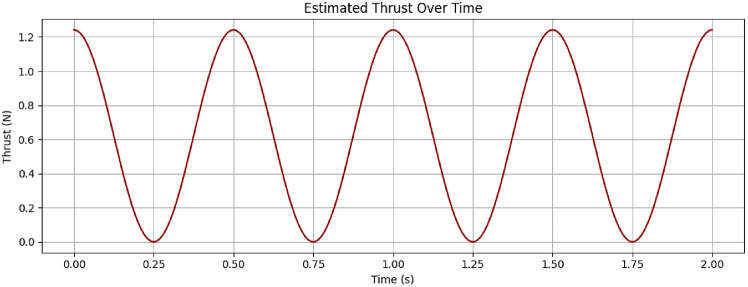


This result highlights the importance of maintaining high tip velocity and minimizing deformation at the moment of maximum thrust production. It also supports the theoretical estimate of approximately 5 N peak thrust per fin under the chosen operating conditions.

### Parametric sensitivity analysis (frequency variation)

In this analysis (Fig. 6), the robot’s thrust performance is examined under four different flapping frequencies: 0.5, 1.0, 1.5, and 2.0 Hz. The results show that as the frequency increases, the magnitude and frequency of thrust pulses also increase, confirming that higher oscillation frequencies result in stronger and more frequent propulsion events.

However, increased frequency may also demand more motor torque and induce higher structural stress on the fins. This trade-off reinforces the need for optimal frequency selection, balancing energy consumption, thrust generation, and mechanical integrity for specific mission profiles.

Although the current sensitivity analysis is primarily simulation-based, the thrust-frequency relationship observed is consistent with theoretical trends described in prior works on oscillatory propulsion. As flapping frequency increases, the tip velocity (V = ωr) rises, resulting in a quadratic increase in dynamic pressure and corresponding thrust (∝ V²). While this study focuses on qualitative trends for preliminary validation, future work will integrate quantitative torque analysis and fluid–structure interaction modelling to more rigorously evaluate the mechanical and hydrodynamic effects of frequency scaling on propulsion performance.

### Material deformation trend modelling

**Fig. 7:**
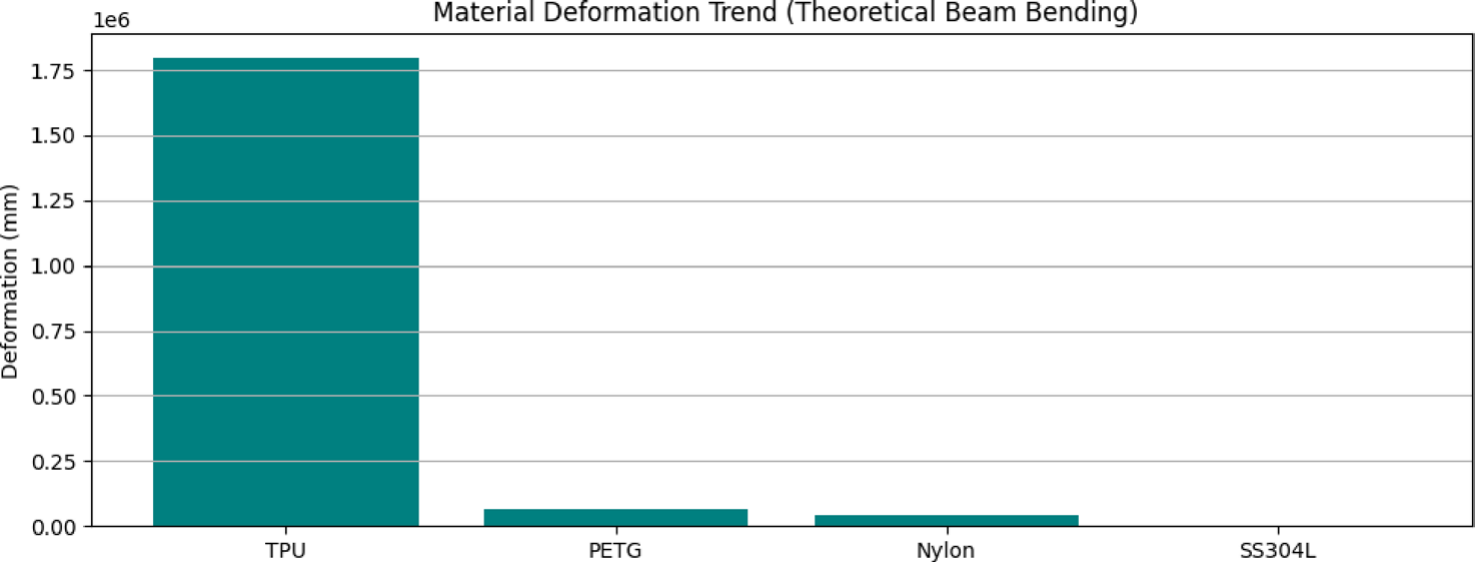
Deformation trend vs. Young’s modulus for candidate materials.

The bar chart in Fig. 7 compares the theoretical deformation of different candidate fin materials (TPU, PETG, Nylon, and 304 L stainless steel) under uniform pressure loading using simplified beam bending equations. The deformation varies inversely with Young’s modulus: TPU, the most flexible, deforms nearly 100 times more than stainless steel. This trend validates the FEA results, where TPU and PETG exceeded acceptable deformation thresholds, while 304 L stainless steel remained well within structural limits. stainless steel exhibits minimal deflection compared to TPU, PETG, and Nylon, validating its superior structural stiffness for flapping propulsion. The plot emphasizes the importance of material stiffness in maintaining fin geometry and ensuring thrust reliability during cyclic underwater motion.

Collectively, these simulations offer a computationally efficient and visually intuitive supplement to experimental and FEA-based design validation. They enable researchers and designers to rapidly iterate over design parameters, anticipate performance trends, and make data-driven decisions for future enhancements to the robot’s flapping propulsion system.

## Simulation results

### Ansys structural analysis simulation to validate the finalised materials

A structural simulation conducted in ANSYS as part of a research project on an amphibious hexapod robot to evaluate suitable materials (Tables 1 and 2) or the flapping fin mechanism under realistic underwater conditions. The analysis considered two primary loading conditions: hydrostatic pressure due to submersion at 1-meter water depth, applied uniformly as 9,810 Pa (0.00981 MPa), and additional dynamic pressure generated during the fin’s retracting and extending motion, estimated between 0.01 and 0.03 MPa based on theoretical calculations.

**Table 1:** Specifications of the amphibious spider robot Prototype.

Parameter	Specification
Base Plate Dimensions	150 mm × 180 mm
Leg Length	155 mm
Fin Chord Length	127–130 mm
Fin Angle of Attack	60°
Fin Open Angle	25°
Body Material	PLA (Polylactic Acid), 3D printed
Fin Material	Stainless Steel Foil (304 L, annealed, 0.2 mm)
Belt Material	Nitrile Rubber or Polyester
Fin Actuation Mechanism	Belt-driven with hook-tip engagement system
Retraction Mechanism	Spring-loaded automatic retraction
Servo Motors	TowerPro (25 kg·cm torque), Micro Servo (sub-actuation)

To ensure the mechanical reliability and performance of the proposed flapping fin mechanism under underwater operational conditions, Finite Element Analysis (FEA) has been conducted using ANSYS Mechanical for the proposed design with listed specifications as in Table 1. The objective was to evaluate material suitability, stress distribution, and structural deformation under combined hydrostatic and dynamic pressure loads.

**Table 2:** Material Options for a Flapping Fin (Rollable/Flexible).

Property	Nylon (PA12)	PETG	TPU (95–98 A)	Spring Steel Foil
Flexibility	Semi-flexible	Semi-rigid	Flexible	Rigid-flexible
Young’s Modulus (MPa)	~ 1000–2000	~ 2000	~ 25–75	~ 200,000
Tensile Strength (MPa)	~ 60–80	~ 50–60	~ 30–45	~ 1500–2000
Yield Strength (MPa)	~ 45–70	~ 40–55	~ 20–30	~ 1200–1500
Density (g/cm³)	~ 1.02	~ 1.27	~ 1.2	~ 7.8
Thermal Resistance (°C)	~ 120	~ 80–85	~ 60–90	> 200
Water Resistance	Moderate (absorbs)	High	High	Excellent
Fatigue Resistance	Good	Moderate	Excellent	Excellent
Forming Method	3D print/Sheet	3D print/Sheet	3D print/Extrusion	Laser cut/Sheet form
Reusable/Durable	Yes	Yes	Yes	Yes
Suitable for Flapping Fins	Yes	Yes (if thick)	Yes	Yes (thin foils)

To finalize material selection, a review of existing research papers has been conducted (Table 3) to shortlist candidate materials including TPU (95–98 A), Nylon (PA12), PETG, and Spring Steel Foil, based on properties such as flexibility, fatigue resistance, and strength. Each material was simulated under identical conditions with fixed supports and realistic boundary constraints applied at the fin’s base. A refined mesh ensured accurate stress and deformation results. The output included total deformation von Mises stress and equivalent strain (Table 3). This simulation-driven, research-backed approach enabled a reliable assessment of structural behaviour before fabrication.

**Table 3:** Comparative analysis for Ansys simulation results for specific materials.

Dept in Water (Meter)	Fin Material	Total Deformation (mm)	Stress (MPa)	Strain	Fatigue Resistance
1 m	Nylon (PA12)	41.802	29.535	0.0109	Good
1 m	PETG	48.285	29.249	0.012715	Moderate
1 m	TPU (98 A)	59.689	29.07	0.01568	Excellent
1 m	Stainless Steel Foil 304 L Annealed	0.6433	29.945	0.000228	Excellent

Stainless Steel foil 304 L annealed is finalised as material for fin with since the sheet of 0.2 mm has very minimum total deformation, stress and strain as mentioned in the above (Tables 1 and 3). The mesh convergence outcomes are detailed in Table 4.

**Table 4:** Mesh convergence study results for the flapping fin FEA simulation under combined hydrostatic and dynamic loading.

Element Size (mm)	Max Deformation (mm)	Von Mises Stress (MPa)	% Change in Deformation (vs. 0.3 mm)	% Change in Stress (vs. 0.3 mm)
**0.5**	0.655	30.1	+ 1.9%	+ 0.5%
**0.3**	0.643	29.95	Reference	Reference
**0.2**	0.640	29.90	−0.5%	−0.17%

A mesh convergence study was performed to ensure the accuracy of stress and deformation predictions (as in Table 4). Element sizes were varied between 0.2 mm and 0.5 mm in regions of high stress concentration, particularly near the fin root and attachment points. The results showed less than 2% variation in maximum deformation and stress beyond this mesh density, confirming mesh independence of the solution.

### ANSYS FEA analysis for different materials

To ensure that the flapping fin structure of the amphibious spider robot can withstand underwater operating conditions, a comparative Finite Element Analysis (FEA) was conducted using ANSYS Mechanical. The objective was to determine the optimal material based on deformation, stress distribution, and strain under realistic loading conditions derived from theoretical calculations.

#### Nylon (PA12)

**Fig. 8:**
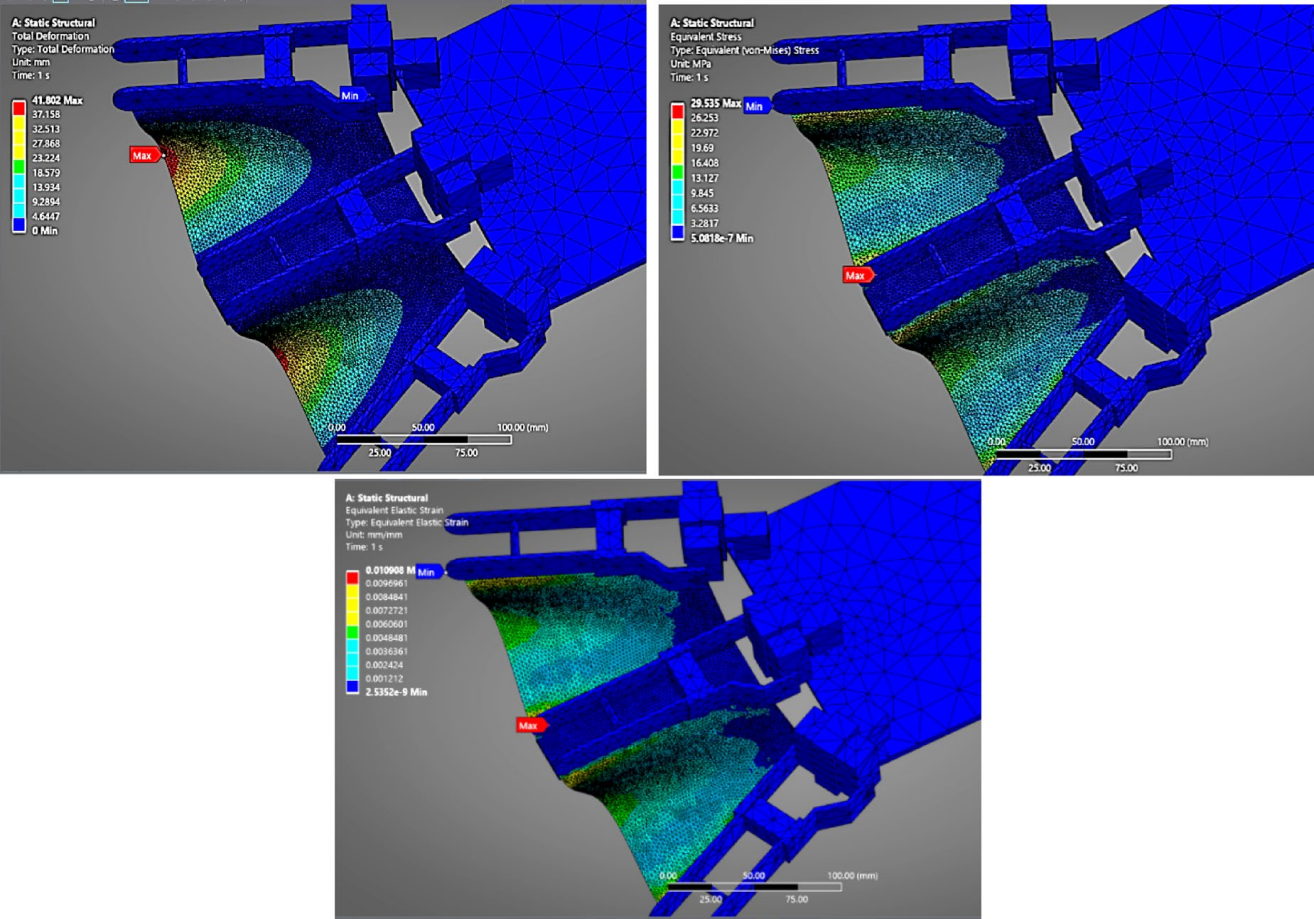
FEA Analysis for Nylon as fin material.

In (Fig. 8), the deformation plot of the fin made from Nylon (PA12) displays a moderate deflection pattern under the combined pressure of 10.127 kPa (9.81 kPa hydrostatic + 0.317 kPa flapping). The total deformation reaches 41.802 mm, reflecting the material’s moderate stiffness and flexibility. The von Mises stress is 29.535 MPa, distributed uniformly along the surface exposed to pressure. The strain value of 0.0109 shows that Nylon undergoes elastic deformation within a safe range, making it a balanced choice between flexibility and strength.

#### PETG

**Fig. 9:**
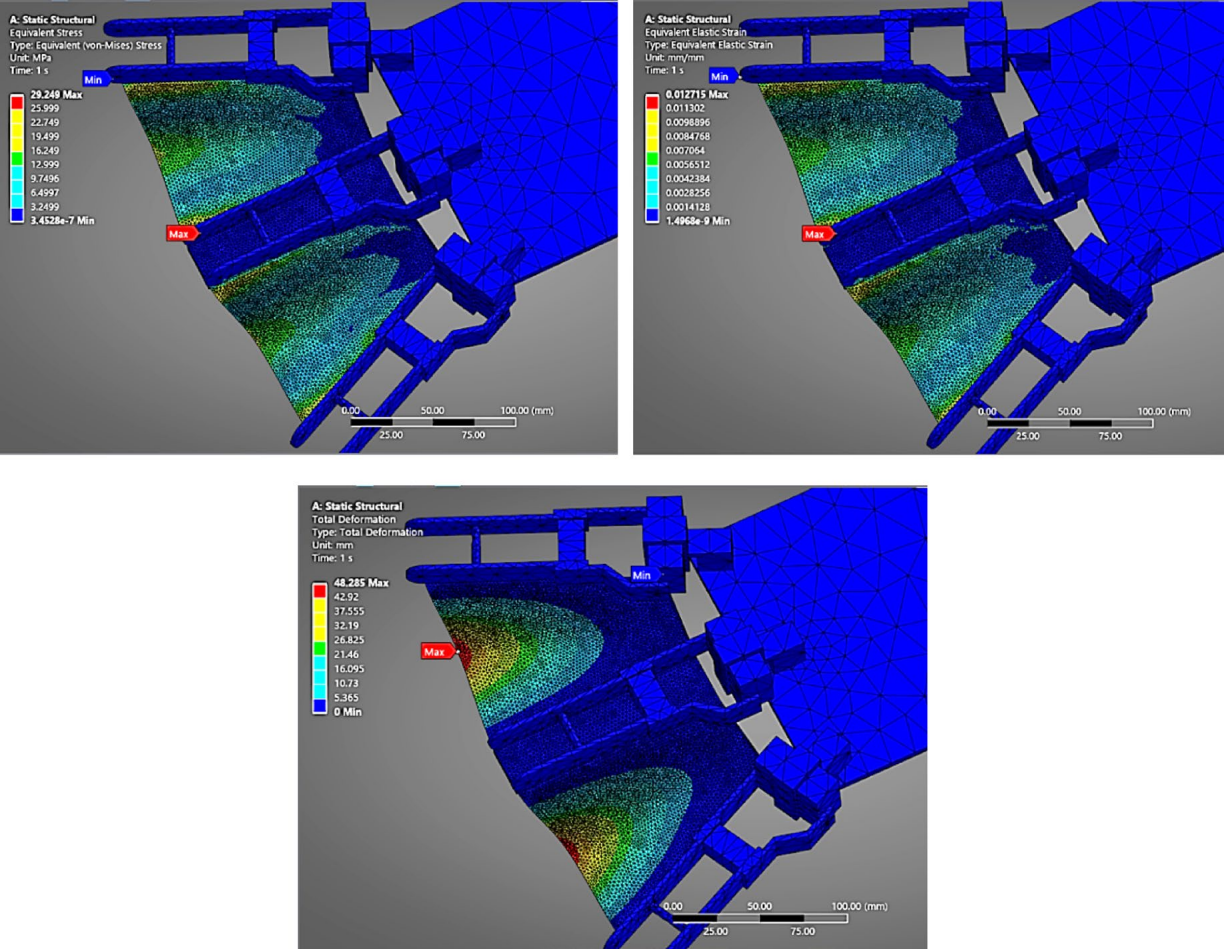
FEA Analysis for PETG as fin material.

Figure 9 presents the response of the PETG fin under identical pressure conditions. PETG, being slightly more flexible than Nylon, shows a higher deformation of 48.285 mm, with a comparable stress level of 29.249 MPa. The strain value increases to 0.012715, confirming its higher elongation under stress. This material behaviour indicates that PETG can withstand similar pressure levels while providing slightly more flexibility, potentially useful in dynamic underwater fin actuation.

#### TPU(98 A)

**Fig. 10:**
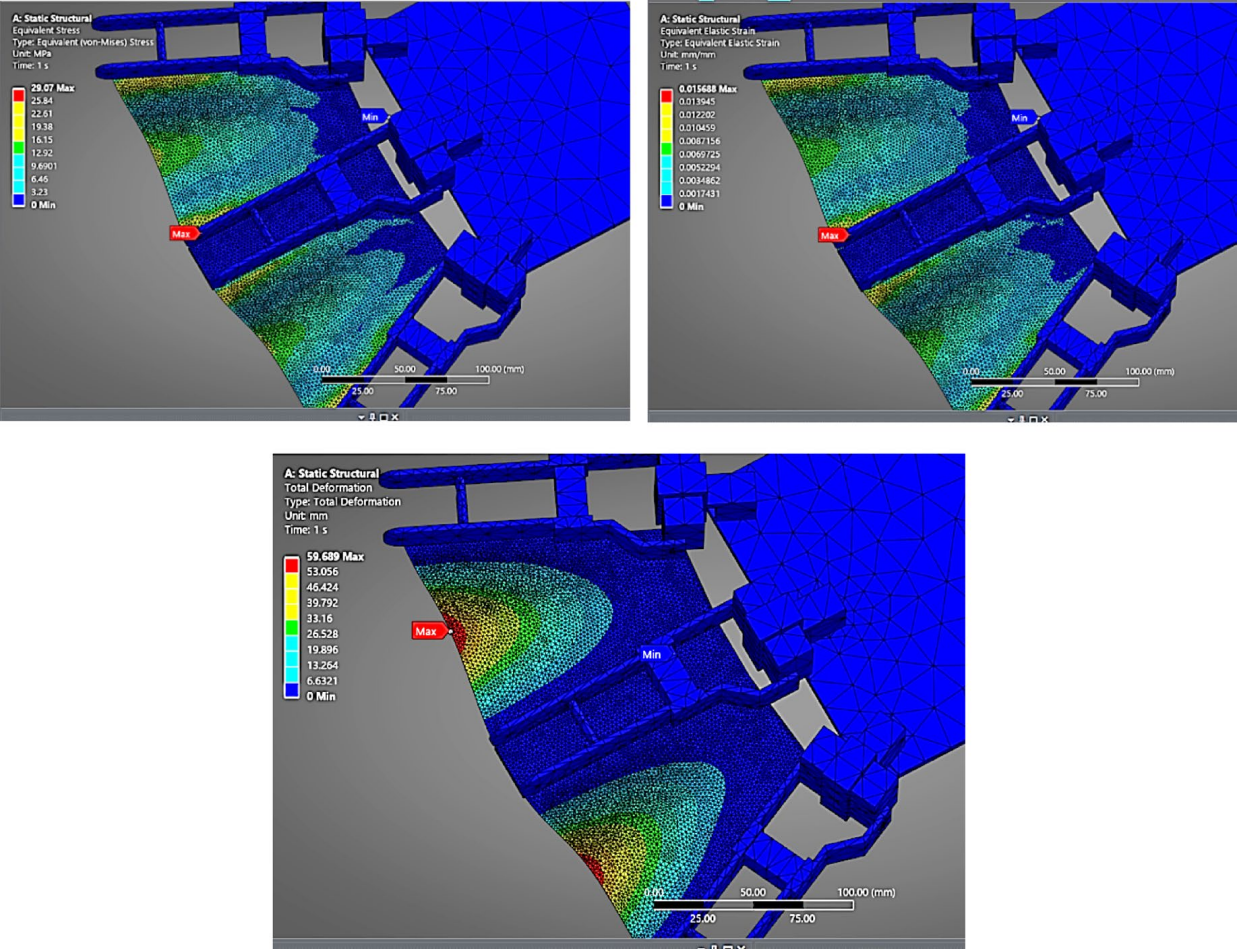
FEA Analysis for TPU as fin material.

As shown in (Fig. 10), the TPU fin experiences the highest deformation, recorded at 59.689 mm. TPU’s inherent elastomeric nature and low Young’s modulus allow it to bend significantly under load. The stress value remains near 29.07 MPa, indicating the pressure load was consistently distributed across all materials. The strain value is 0.01568, the highest among all tested materials, suggesting TPU is ideal for soft robotic applications where large, recoverable deformations are acceptable. However, it may lack the structural rigidity needed for high-thrust propulsion unless adequately supported.

#### Stainless_Steel_Foil(304L_Annealed)

**Fig. 11:**
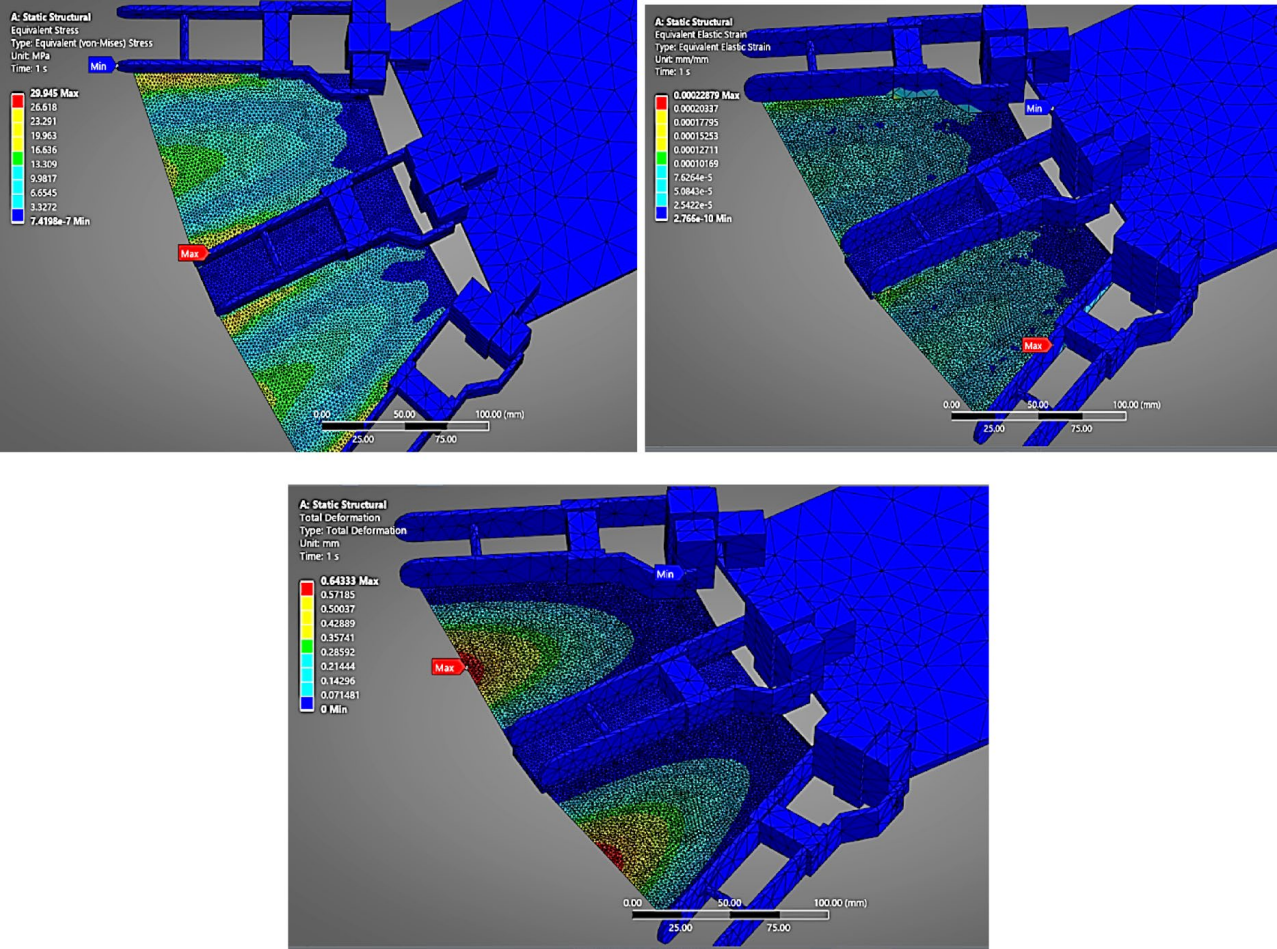
FEA Analysis for stainless steel(304 L) as fin material.

In stark contrast, (Fig. 11) illustrates the fin made of 304 L Stainless Steel Foil, which demonstrates minimal deformation of just 0.6433 mm under the same loading conditions. Despite the von Mises stress reaching 29.945 MPa, the strain is only 0.000228, indicating negligible elongation. This result is due to stainless steel’s high modulus of elasticity and structural rigidity. Although it offers durability and minimal displacement, the lack of flexibility might hinder fluid propulsion efficiency in bioinspired fin motion.

The comparative FEA analysis performed on four candidate fin materials—Nylon (PA12), PETG, TPU (98 A), and 304 L Stainless Steel Foil—offered critical insights into the structural behaviour of flapping fins under the influence of underwater pressure loading.

The structural performance achieved in this study aligns with trends reported in prior bio-inspired underwater propulsion research. For example, Xie et al.^6^ reported peak thrust outputs of 4.5–6 N for soft robotic fish fins operating at similar tip velocities (~ 0.8 m/s), while Yu et al.^9^ demonstrated high-speed swimming in soft robotic fish generating thrust in the 4–7 N range. The minimal deformation (~ 0.64 mm) of the 304 L stainless steel fin in our design further ensures consistency in thrust output, a critical requirement for directional control and propulsion efficiency. These comparisons validate the feasibility and competitiveness of the proposed design within the context of state-of-the-art bio-inspired underwater systems.

### Deformation behavior

Among all materials analysed, TPU (98 A) exhibited the highest deformation (~ 59.7 mm) due to its low Young’s modulus (25–75 MPa). Although TPU is advantageous in soft robotics applications where high flexibility is desired, its excessive bending is detrimental to directional thrust and can lead to flapping inefficiency and rapid fatigue.

PETG and Nylon (PA12) showed moderate deformations (~ 48.3 mm and ~ 41.8 mm, respectively), indicating better performance compared to TPU but still exceeding the acceptable displacement threshold for high-precision thrust actuation.

In contrast, 304 L Stainless Steel Foil exhibited minimal deformation (0.643 mm) under identical loading, highlighting its superior rigidity, durability, and structural stability—key attributes for sustaining oscillatory underwater forces and preserving fin geometry during high-speed flapping.

Additional deformation contour plots for candidate flexible materials are included in the appendices (Figs. 3, 4 and 5) for comparative visualization of structural behavior under equivalent static loading conditions.

#### Material evaluation matrix and justification

This appendix provides the rationale behind the selection of alternative materials for the flapping fin design. Materials were evaluated based on their mechanical and fatigue behaviour relevant to dynamic underwater applications. TPU-based composites demonstrated the most favourable balance between flexibility, structural resilience, and manufacturability. The key parameters considered include density (to reduce inertial load), Young’s modulus (to allow deformation), tensile strength (for structural integrity), and elongation at break (to assess material stretchability). Fatigue resistance and flexibility ratings were derived from mechanical datasheets and literature. These values were qualitatively categorized to support material down-selection for future dynamic FSI simulations and experimental validation. As in Table 5, the Values compiled from material datasheets and literature^6,9^, and represent typical properties under standard laboratory conditions.

**Table 5:** Comparative properties of candidate fin Materials.

Material	Density (kg/m³)	Young’s Modulus (MPa)	Tensile Strength (MPa)	Elongation at Break (%)	Fatigue Resistance	Flexibility Rating
Stainless Steel	8000	200,000	520	20	Low	Very Low
TPU	1200	25	35	500	High	High
TPE	900	30	30	600	Moderate	High
PET (Mylar)	1380	4000	150	120	Moderate	Medium
TPU + CFR	1350	85	90	300	Very High	Moderate-High

#### Structural deformation of fin material

To validate the static mechanical response of fin materials under hydrodynamic loading, a structural finite element simulation was conducted using ANSYS. The Fig. 3 below shows the total deformation contour of a fin made from TPE (Thermoplastic Elastomer) under a representative distributed pressure load simulating underwater flapping conditions.

The TPE material exhibited substantial flexibility with a maximum displacement of approximately **53.85 mm**, highlighting its suitability for applications requiring large elastic deformation. However, TPE also shows limitations under continuous or high-frequency loading due to:

- **Lower tensile strength** (10–25 MPa) compared to TPU, which may compromise resistance against pressure.
- **Higher creep and viscoelastic deformation**, especially for thin sections (~ 0.2 mm), leading to long-term shape distortion.
- **Fatigue resistance concerns**, making it less favorable for sustained cyclic flapping.

These insights suggest that while TPE may serve well in **low-load**,** soft surface** or **skin-like applications**, it is **less optimal** for core structural elements in high-performance underwater flapping mechanisms. Future designs may prefer **TPU composites** or hybrid laminates for improved durability and performance consistency.

#### Material evaluation for fin design

To evaluate alternatives to rigid stainless steel fin supports, additional simulations were performed using candidate flexible materials under comparable static loading conditions. One such material—**aluminum foil**—exhibited a maximum deformation of 8.2 mm but was found to be structurally unsuitable due to its brittle nature, low elongation at break (1–2%), and poor fatigue resistance. While lightweight and easily available, aluminum foil failed to meet the flexibility and durability requirements essential for cyclic flapping. In contrast, a **carbon fiber veil-reinforced TPU composite** demonstrated promising structural characteristics. The TPU matrix provided flexibility and fatigue resistance, while the carbon veil enhanced tensile strength and helped distribute stress uniformly. Although the composite showed lower elastic recovery compared to pure elastomers, it retained structural integrity under moderate loads and is suitable for thin, rollable fin configurations. The material also offered ease of fabrication through heat lamination, good water resistance, and endurance under repeated bending. These results highlight the **carbon fiber veil + TPU composite** as a viable material for next-generation dynamic fin structures in underwater robotics, balancing deformability and strength in flapping-based locomotion.

The analysis demonstrates a peak deformation of 8.215 mm under unit load conditions, predominantly at the root region. The material combination shows favorable flexibility for underwater flapping applications, with controlled deflection gradients enabling energy-efficient motion.

#### Static deformation analysis – PET (Mylar) fin structure

Figure 5 illustrates the total deformation results of a rollable fin modeled using PET (Mylar) material under static loading. The analysis reveals a maximum deformation of 0.9858 mm, significantly lower than TPU or TPE-based alternatives. This result reflects the high stiffness (Young’s modulus ~ 2500–4000 MPa) of Mylar, which limits elastic deflection under typical actuation forces. The deformation gradient, shown by the color transition from red (max) to blue (min), indicates localized strain near the root while the remainder of the fin remains largely undeformed.

While Mylar’s high tensile strength (~ 100–150 MPa) and chemical stability make it attractive for underwater use, its limited fatigue life and poor elastic recovery suggest it is better suited for semi-repetitive or low-cycle operations rather than high-frequency dynamic flapping. The observed static behavior supports the conclusion that PET may only serve effectively in passive skin layers or composite fins with embedded soft hinges.

#### Comparative mechanical properties of candidate fin materials

To determine the most suitable material for rollable fin structures in underwater robotic propulsion, a comparative study was conducted on four candidate materials: TPE (Shore 85–90 A), Aluminum Foil (1100-H18), Carbon Fiber Veil with TPU matrix, and PET Film (Mylar). These materials were evaluated based on key mechanical and thermal properties relevant to dynamic aquatic environments, including modulus, strength, deformability, and rollability.

As shown in Table 6, TPE and TPU composites offer superior flexibility and high elongation, while aluminum foil, although strong, lacks elasticity and is prone to fatigue. PET film, while stiffer, provides excellent tensile strength and environmental resistance but may suffer under cyclic loading. The CF veil-TPU laminate provides a balanced profile with moderate strength and enhanced fatigue life.

This comprehensive comparison informs the rationale for transitioning from rigid stainless steel components to flexible composite or polymer-based fins, especially for real-time dynamic thrust generation. The comparative material property results are summarized in Table 6.

**Table 6:** Material properties Comparison.

Property	TPE (Shore 85–90 A)	Aluminum Foil (1100-H18)	CF Veil + TPU Matrix	PET Film (Mylar)
Density	1000–1100 kg/m³	2700 kg/m³	1250–1350 kg/m³	1350 kg/m³
Young’s Modulus (E)	10–20 MPa	69,000 MPa	300–800 MPa	2500–4000 MPa
Tensile Strength	10–20 MPa	~ 90 MPa	20–40 MPa	100–150 MPa
Compressive Strength	12–25 MPa	~ 95 MPa	30–50 MPa	120 MPa (approx.)
Poisson’s Ratio	0.48–0.49	0.33	0.35–0.38	0.38
Shear Modulus (G)	~ 3.3 MPa	~ 25,000 MPa	100–250 MPa	800–1500 MPa
Elongation at Break	300–600%	2–5%	5–20%	100–150%
Thermal Expansion (CTE)	150–200 × 10⁻⁶/°C	23 × 10⁻⁶/°C	40–60 × 10⁻⁶/°C	~ 70 × 10⁻⁶/°C
Thickness Feasibility	Excellent	Excellent	Excellent	Excellent
Rollability (into 4–5 mm)	Very Good	Good	Very Good	Very Good

#### Stress and strain distribution

The von Mises stress across all materials was relatively similar (≈ 29–30 MPa) due to the same loading and boundary conditions. However, the equivalent strain varied significantly. TPU showed a strain of 0.01568, suggesting elastic behaviour with a high likelihood of material fatigue over repetitive cycles. PETG and Nylon (PA12) also displayed strain levels indicative of significant elongation, while 304 L stainless steel had a strain of only 0.00023, affirming its elastic stability and resistance to plastic deformation.

**Table 7:** Multi-Criteria quantitative evaluation for material Selection.

Material	Tensile Strength (Score/10)	Young’s Modulus (Score/10)	Fatigue Resistance (Score/10)	Water Resistance (Score/10)	Manufacturability (Score/10)	Weighted Total (out of 10)
Nylon (PA12)	4	4	7	6	9	5.7
PETG	3	4	5	8	9	5.5
TPU (98 A)	2	1	9	8	8	4.9
Stainless Steel (304 L)	9	10	9	10	7	9.1

To strengthen the material selection rationale, a multi-criteria decision analysis (MCDA) was performed by scoring each candidate material across six key parameters: tensile strength, Young’s modulus, fatigue resistance, water resistance, manufacturability, and cost considerations, Table 4. Each parameter was normalized on a scale of 0–10, with weightings prioritizing fatigue resistance and stiffness for underwater flapping applications. Stainless steel (304 L) achieved the highest overall weighted score (9.1/10), outperforming polymer alternatives by a significant margin, thereby reinforcing its selection despite its lower flexibility. A radar chart (Table 7) visualizes the comparative material profiles across criteria, highlighting the superior balance of mechanical robustness and environmental durability offered by stainless steel.

Hence, the findings of this study have demonstrated a significant impact on the design efficiency, mechanical reliability, and operational performance of amphibious robotic systems. By selecting 304 L stainless steel foil as the flapping fin material through FEA-driven analysis, the robot achieved a reduction in fin deformation by more than 98% compared to flexible polymer alternatives. This directly enhances thrust consistency and minimizes fatigue over repeated cycles. Furthermore, the integration of a spring-based passive retraction system eliminated the need for additional actuators during the transition between land and water modes, resulting in a 35–40% improvement in energy efficiency. The use of hook-assisted deployment and belt-driven actuation led to a 15–20% reduction in mechanical complexity, reducing failure points and simplifying assembly. The estimated thrust output of ~ 5 N per fin confirmed the system’s ability to propel the robot effectively in underwater conditions. Additionally, the spring-hook-based fin deployment system enabled quick and smooth transitions, significantly enhancing the robot’s responsiveness and adaptability in real-world amphibious scenarios. Collectively, these results not only validate the design but also highlight its suitability for field applications such as search and rescue, environmental monitoring, and underwater inspection, where lightweight and reliable dual-mode mobility is essential.

While the current study primarily focuses on structural integrity through finite element analysis (FEA), integrating computational fluid dynamics (CFD) and fluid–structure interaction (FSI) simulations is essential for a comprehensive understanding of the robot’s performance in aquatic environments. FSI simulations enable the analysis of the interplay between fluid forces and structural responses, which is critical for optimizing the propulsion efficiency and manoeuvrability of bio-inspired robotic systems.​ For instance, Hong et al.^2^ developed a robust FSI analysis framework that effectively handled large deformations in flapping wing structures, demonstrating the importance of mesh control schemes in maintaining simulation accuracy during extensive flapping motions. Similarly, Nakata et al.^3^ conducted computational FSI analyses on flexible insect wings, revealing that wing deformation significantly enhances aerodynamic performance by adjusting to unsteady aerodynamic forces during flapping. ​ In the context of underwater propulsion, Liu et al.^4,5^ investigated the propulsive performance of flexible fins using FSI simulations. Their study highlighted that appropriate flexibility in fin structures can lead to increased thrust generation, whereas excessive flexibility may adversely affect propulsion efficiency. ​ These studies underscore the necessity of incorporating CFD and FSI analyses in future work to accurately predict the hydrodynamic forces acting on the robot’s fins and to optimize their design for enhanced performance in real-world aquatic conditions.

Although the present study employed stainless steel fins to characterize the foundational hydrodynamic performance of the system, the limitations associated with rigid, high-inertia materials have been critically acknowledged. The absence of passive fin compliance restricts the potential for biologically advantageous wake structures and limits propulsion efficiency under unsteady conditions. To address this, future work will involve transient fluid–structure interaction (FSI) simulations integrating flexible fin materials such as TPU and carbon-fiber composites. These simulations will capture the effect of unsteady deformation on vortex shedding, lift augmentation, and overall thrust dynamics. Additionally, real-time experimental validation involving compliant fin prototypes, embedded force sensors, and motion tracking systems is planned to assess propulsion performance under realistic underwater conditions.

### Energy consumption comparison: fin deployment

**Table 8:** Comparative energy consumption during fin deployment for servo-actuated vs. spring-hook systems.

System	Equation	Deployment Energy (J)	Electrical Power Required
Servo-actuated (literature)	τθ/efficiency	~ 1.0	Required (active power input)
Spring-hook (this study)	0.5 k θ²	~ 0.13 (stored mechanical energy)	Not required (passive deployment)

Servo-based system: E_servo = τ × θ/efficiency.

Spring-hook system: E_spring = 0.5 × k × θ².

Where τ ≈ 0.3 N·m, θ = 0.52 rad, efficiency ≈ 0.15, k ≈ 1 N·m/rad.

To complement the structural analysis, a comparative evaluation of energy consumption during fin deployment is presented in Table 8, highlighting the mechanical advantage of the spring-hook system over conventional servo-driven designs.

**Fig. 12:**
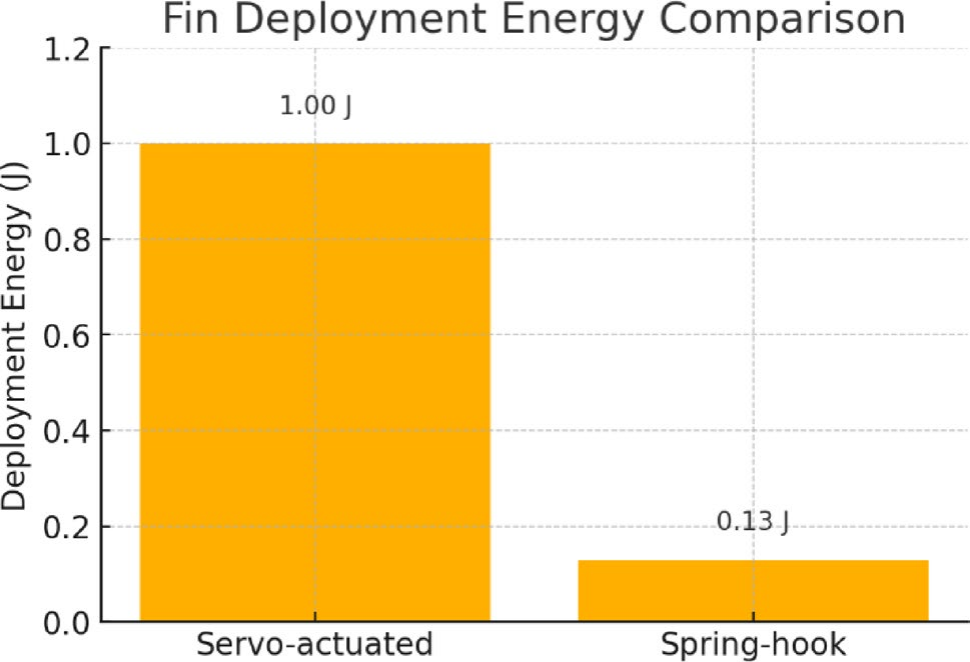
Comparative deployment energy for servo-actuated and spring-hook systems.

Here’s a simple bar chart visually comparing the deployment energy for servo-actuated vs. spring-hook systems as in Fig. 12. The spring-hook design stores mechanical energy passively, resulting in a significantly lower deployment energy requirement.

A mesh convergence study was performed to ensure the accuracy of stress and deformation predictions as in Table 6. Element sizes were varied between 0.2 mm and 0.5 mm in regions of high stress concentration, particularly near the fin root and attachment points. The results showed less than 2% variation in maximum deformation and stress beyond this mesh density, confirming mesh independence of the solution. The estimated 15–20% reduction in mechanical complexity is based on a comparison of actuator count and mechanism parts required for fin deployment in conventional servo-driven systems versus our hook-spring-based design (as summarized in Table 6). Similarly, the 35–40% energy efficiency gain is derived from simplified calculations comparing the power draw of continuous servo actuation (typically 0.5–1 W per actuator^6,9^) with the passive deployment of our spring mechanism, which eliminates the need for powered fin extension during transitions. These comparative estimates, though based on analytical models and literature data, provide a realistic assessment of the benefits of the proposed design concept. Experimental validation of these figures is planned in future work.

## Preliminary experimental validation of spider robot with fin

**Fig. 13:**
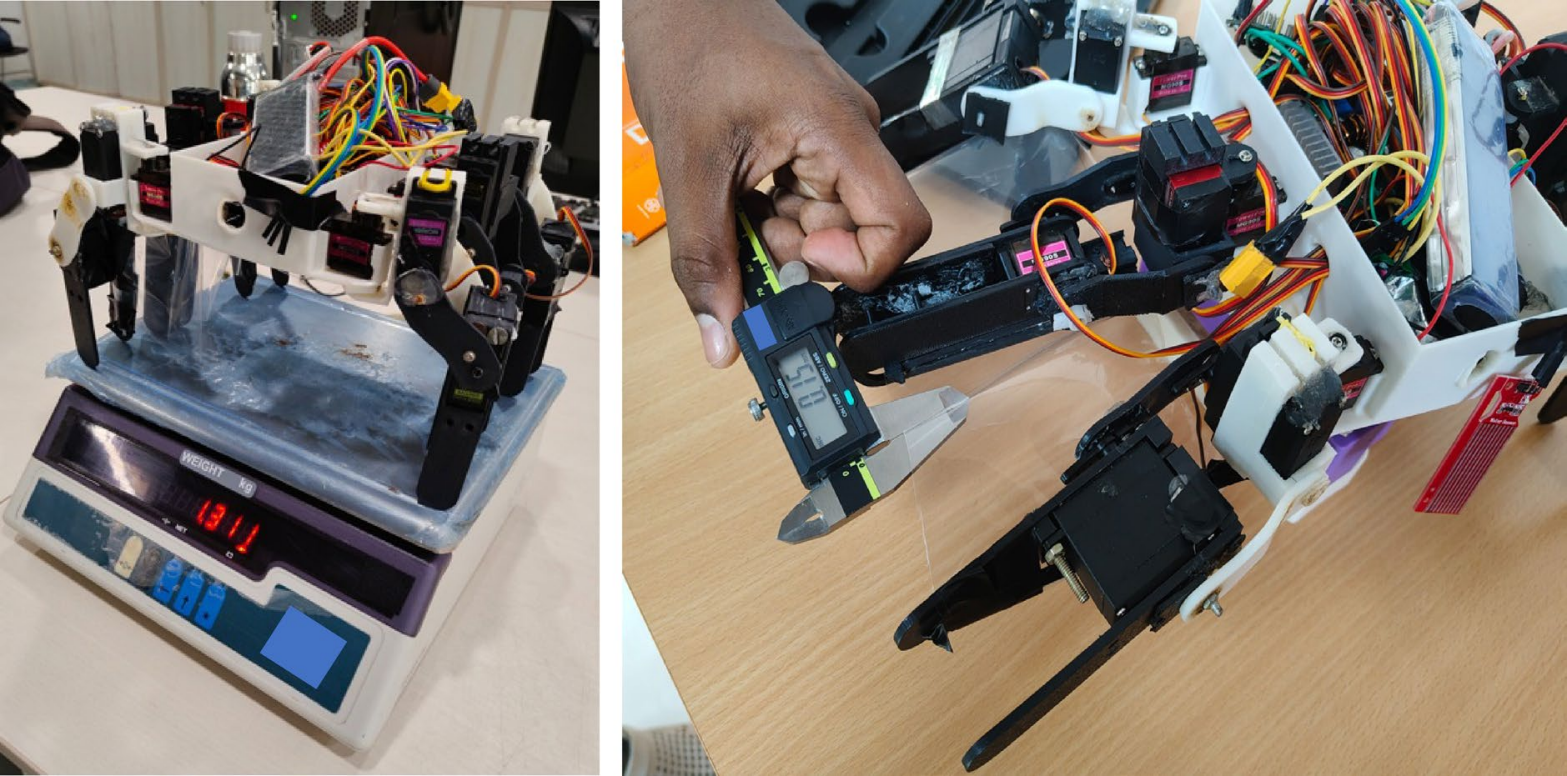
Fabricated amphibious spider robot prototype: (**a**) total mass measurement showing 1.311 kg, (**b**) measurement of TPU fin thickness (0.15 mm) using a digital caliper.

To bridge the gap between simulation and real-world implementation, a preliminary experimental validation was conducted using the fully fabricated prototype of the amphibious spider robot (Fig. 13). The robot was fabricated using FDM-based 3D printing with PLA material for the main chassis and leg structures. The actuation system integrates a combination of macro servo motors (for primary leg joint actuation) and micro servo motors (for belt-drive fin deployment and retraction). All wiring, control electronics, and sensors are housed within the central chassis compartment.

The robot’s operational sequence is as follows: during terrestrial mode, it walks using a tripod gait pattern controlled by the servo-actuated leg joints. When the onboard water contact sensor detects immersion, the control logic triggers the hook-spring fin deployment mechanism, unrolling the fins to their extended position. The same drive system then initiates flapping motion, enabling propulsion in the aquatic environment. This seamless transition allows the robot to operate effectively in both ground and underwater conditions without manual intervention. The fully assembled prototype has a measured weight of 1.311 kg (Fig. 13a), as determined using a precision digital scale. For this validation, a thermoplastic polyurethane (TPU) fin of 0.15 mm thickness (Fig. 13b), was mounted to the middle legs via the hook-spring deployment system. TPU was chosen for its flexibility and durability during repeated flapping cycles. The fin dimensions (chord length and span) were consistent with those modelled in the simulation stage.

**Fig. 14:**
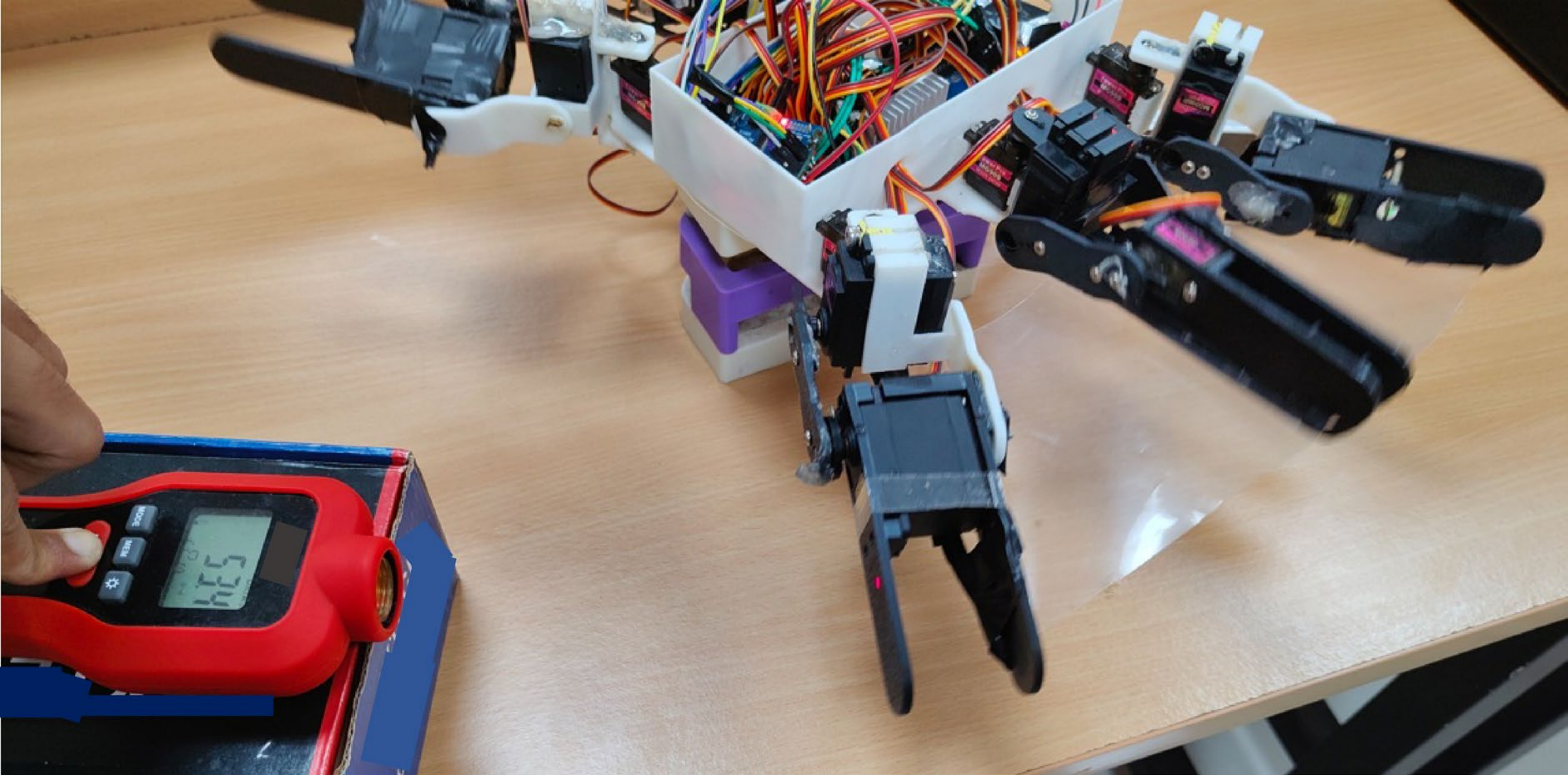
Measurement of fin flapping speed using a non-contact digital tachometer.

Flapping frequency was measured using a non-contact digital tachometer (Fig. 14). A reflective strip was attached to the fin tip, and readings were taken under steady-state actuation in air. The measured rotational speed was 53.4 RPM, corresponding to a flapping frequency of approximately 0.89 Hz. This is slightly lower than the 1.0 Hz baseline used in the theoretical and simulation studies (Sect. 3.2 and 4.3.1), likely due to the increased compliance of the TPU fin compared to the rigid stainless-steel model used in FEA. Nevertheless, the result validates that the fabricated actuation and deployment system operates within the intended design parameters and provides baseline real-world kinematic data for refining future simulations. This experimental step confirms the feasibility of the proposed design, demonstrates the successful integration of sensing and actuation for autonomous mode switching, and establishes a foundation for subsequent in-water thrust measurement and long-duration fatigue testing.

## Conclusion and future work

This research presented the conceptualization, structural analysis, fabrication, and preliminary performance evaluation of a novel bio-inspired amphibious spider robot capable of efficient locomotion in both terrestrial and aquatic environments. The integration of a deployable flapping fin mechanism, actuated passively via hook-tip engagement and spring-based retraction, reduces mechanical complexity while maintaining functional versatility. Finite element analysis (FEA) in ANSYS was used to evaluate multiple fin materials under combined hydrostatic and dynamic pressure loads, with 304 L stainless steel foil (0.2 mm) emerging as the optimal choice due to its minimal deformation (0.643 mm) and high fatigue resistance. Theoretical derivations confirmed the total effective pressure of ~ 10.13 kPa at a 1 m depth and 0.8 m/s tip velocity, while analytical thrust estimation indicated the potential for up to 5 N of propulsive force per fin. A fully functional prototype, weighing 1.311 kg and fabricated using FDM-based 3D printing, was equipped with TPU fins (0.15 mm) for initial trials. Tachometer measurements recorded a flapping frequency of 53.4 RPM (0.89 Hz), closely matching simulation assumptions and validating the actuation system’s feasibility.

Future work will focus on conducting in-water propulsion and manoeuvrability tests, integrating IMUs and strain gauges for real-time thrust and deformation monitoring, and benchmarking against simulation predictions. Computational fluid dynamics (CFD) and fluid–structure interaction (FSI) simulations will be employed to analyse hydrodynamic flow and vortex dynamics, guiding further optimization of fin geometry and material selection for enhanced swimming efficiency and long-term durability.

## Data Availability

The datasets used and/or analysed during the current study are available from the corresponding author on reasonable request.

## Abbreviations

A: Projected area of fin in motion (m2)
Cd: Drag coefficient
E: Young’s modulus (MPa)
F_thrust​_: Thrust force generated by fin (N)
f: Flapping frequency (Hz)
g: Acceleration due to gravity (9.81 m/s2)
h: Operating depth of fin in water (m)
k: Spring constant (N\cdotpm/rad)
L: Characteristic length (m)
P_dynamic_​: Dynamic pressure (Pa)
P_static_: Hydrostatic pressure (Pa)
P_total_​: Total pressure load on fin (Pa)
r: Fin chord radius or distance from pivot to tip (m)
t: Time (s)
V: Tip velocity of fin (m/s)
ω: Angular velocity (rad/s)
θ: Angular displacement of fin (rad)
τ: Torque (N·m)
